# COVID-19 Response and Complex Emergencies: The Case of Yemen

**DOI:** 10.1017/dmp.2020.271

**Published:** 2020-07-27

**Authors:** Osama Ali Maher, Giuseppe Pichierri, Gabriele Farina, Catello M. Panu Napodano, Saverio Bellizzi

**Affiliations:** Division of Water Resources Engineering, Lund University, Lund, Sweden; Kingston Hospital NHS Foundation Trust, Microbiology Department, Kingston upon Thames, United Kingdom; University of Sassari, Sassari, Italy; Medical Epidemiologist, Independent Consultant, Geneva, Switzerland

**Keywords:** emergency preparedness, epidemics, pandemics

In countries undergoing complex emergencies, free movement, and a number of security issues can deeply affect implementation of any of the traditional social distancing measures deployed during outbreaks. Primarily, internal conflicts are an obstacle toward having a centralized outbreak strategy without the acceptance of all parties involved in a conflict. As a matter of fact, this affects timely declaration of official cases in parts of the country, as well as credibility on numbers of reported cases by conflicting parties.

One striking example is represented by the situation in Yemen, conflict-torn since 2015. Despite the recent declaration of confirmed coronavirus disease (COVID-19) cases, the country is fighting a cholera outbreak that started in 2017 and from which is still reporting suspected cases.

When the COVID-19 outbreak started to be a reality in many countries around the world, the threat, represented by the movement of people across countries, might have led to a false sense of security; in Yemen, the main points of entries to the country are closed, and free movement within the country is not possible in many areas.

The possibility of imported cases has soon proven dangerous since not all movements of population are regulated, and the compromised security situation made the control of borders not handled by a single authority. This adds to the issue of an extremely fragile health system.

**FIGURE 1:**
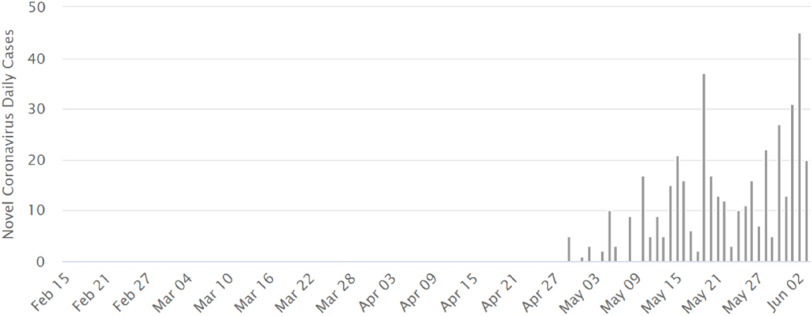
Number of Confirmed COVID-19 Cases in Yemen Until June 4.

The first confirmed COVID-19 case in Yemen was reported on April 28 (Figure 1), well after the virus had been reported in the region and in the neighboring countries, such as Oman and the Kingdom of Saudi Arabia **(**KSA).^1^

Since then, daily cases have been on the rise and most probably the numbers are far from representing the real extent of the outbreak. Until June 4, the total number of cases were 453 with 103 deaths, which means a case fatality rate (CFR) of 27%. This high CFR is most probably due to the extremely low number of tests performed, only 120 tests.^1^

There are no clear strategies in the country at present, and, realistically, there does not seem to be any hope in having a centralized approach toward the control of the outbreak. Movement restrictions, a weak health system, and a complex political situation are all strong indicators that the situation in Yemen might be more catastrophic than what we may anticipate.

Social distancing can be impossible to achieve in war zones. At the same time, assuming that a stronger focus on a dramatic health situation would facilitate a peace process is indeed merely an assumption, as this did not seem to make any impact in recent medical emergencies, like the aforementioned cholera outbreak.

What may perhaps be useful for the time being is accurately looking back at experiences of other countries in a similar situation. Complex emergencies and outbreak control should be considered on a case-by-case basis. It would help dramatically to have a political analysis of the situation on the ground and what the existing constraints are in order to potentially escalate appropriate interventions. This is not impossible to achieve since there are many lessons learned from the past. The main focus should be in ensuring that such a dramatic public health emergency may act as a catalyst for peace in a real pragmatic way, which could be a leading example in conflict scenarios.
